# Pharmaceuticals and personal care products in water and wastewater: a review of treatment processes and use of photocatalyst immobilized on functionalized carbon in AOP degradation

**DOI:** 10.1186/s13065-020-00714-1

**Published:** 2020-10-22

**Authors:** Adewumi Olufemi Oluwole, Elizabeth Oyinkansola Omotola, Olatunde Stephen Olatunji

**Affiliations:** School of Chemistry and Physics, University of KwaZulu-Natal, Westville, Durban, 4000 South Africa

**Keywords:** Ppcps, Wastewater, WWTPs, AOPs, Heterogeneous photocatalysis, Photocatalyst immobilized, Activated carbon/graphene/carbon nanotubes solid support, PPCPs degradation, Removal efficiency

## Abstract

The presence of emerging contaminants such as pharmaceutical and personal care products in many aqueous matrices have been reported. One of such matrix is streams of wastewater, including wastewater treatment plants inflows and outflows and wastewater flow by-passing wastewater treatment plants. Their persistence arises from their resistant to breakdown, hence they may remain in the environment over long time, with a potential to cause adverse effects including endocrine disruption, gene toxicity, the imposition of sex organs, antibiotic resistance and many others in some aquatic organisms exposed to arrays of residues of pharmaceutical and personal care products. Among the treatment techniques, advanced oxidation processes have been reported to be a better technique through which these PPCPs can be degraded in the WWTPs. Heterogeneous photocatalysis using various photocatalyst immobilized on solid support such as activated carbon, graphene and carbon nanotubes in AOPs have been shown to be a viable and efficient method of PPCPs degradation. This is because, the performance of most WWTPs is limited since they were not designed to degrade toxic and recalcitrant PPCPs. This review highlight the occurrence, concentration of PPCPs in wastewater and the removal efficiency of heterogeneous photocatalysis of TiO_2_ immobilized on solid supports.

## Introduction

The expansion in the world’s population, economic development, the industrial revolution, and climate change has led to an increase in the quantity of waste generation as well as an increase in the introduction of specific categories of compounds referred to as emerging contaminants, into the environments. The U.S. Environmental Protection Agency (EPA) described “emerging contaminants” as chemicals or materials which were previously not listed for routine monitoring, characterized by a perceived potential or real threat to human and environmental health, and lacks published health standard, environmental protection laws, and regulations [1]. According to the U.S. Geological Survey (USGS), emerging contaminants are “any synthetic or naturally occurring chemical or any microorganism that is not usually investigated in the environment but can find its way into the environment and cause known or suspected adverse ecological and/or human health effects”. Hence, there is an urgent need to develop methods for the removal of these emerging contaminants from the environment.

Emerging contaminants can be classified into several groups, which include; perfluorinated compounds (PFCs), pharmaceuticals and personal care products (PPCPs), brominated flame retardants (BFRs), microplastics and toxins from harmful algal blooms, etc. [2, 3]. Concerns about the potentially adverse effects of these substances on human and ecosystem health are arising from their persistence, environmental accumulation tendency, exposure potential, bioaccumulation/biomagnification potential, and bioactivity. These contaminants may, therefore, be prioritized for regulation, depending on the information pool from monitoring data regarding their occurrence, their toxicity, potential health effects, and public perception.

A significant index factor of public and community health is access to good quality water, which is imperatively crucial for all life forms. The pollution of the water system may result in an outbreak of water-borne diseases, which can have acute or long-term health implications and even death. Humans and animals are susceptible to these diseases because polluted water is mostly the exposure pathway to infectious pathogens and carcinogenic organic and inorganic contaminants. For instance, while the dissolution of active pharmaceutical ingredients (APIs) in aqueous matrices is variable; they are primarily water-soluble and have such properties that allow them to permeate membranes and persist in the biological systems. The inherent properties of APIs, however, raise questions about their environmental risk, the potential for accumulation, and bioactivity within an aquatic environment [4]. As with administered dosages on target organisms, pharmaceuticals are capable of altering biochemical and physiological processes in many non-target organisms. Hence, their capacity to induced undesirable consequences on native and peripatetic species of organisms exposed to APIs contaminated environment.

Although the presence of PPCPs in the environment has been noticed for some years now, the full extent of their presence and the risk associated with their presence in the environment has not been fully established. Also, the removal of these contaminants in wastewater treatment (WWTPs), before their discharge into surface water has proven to be difficult due to their low occurrence concentrations, and the challenges arising from analyzing them [5]. Therefore, there is a need to modify and upgrade the existing WWTPs to be able to resolve or remove these contaminants. The conventional wastewater treatment methods which include; physical, chemical or combination of physicochemical process, involving the following: filtration, flocculation, coagulation process, sedimentation, biological processes, membrane filtration, chlorination, adsorption process via activated carbon (AC), carbon nanotubes (CNTs), graphene oxide (GO), ozonation, photo-catalysis ultraviolet irradiation, ultra-sonication, and others. Unfortunately, these methods cannot adequately remove organic pollutants from wastewater. In addition to the processes being chemically and operationally intensive, they require large systems, infrastructure, and engineering expertise, which make them burdensome, ineffective, time-consuming, and costly [6, 7].

Moreover, the use of nano-adsorbents in the removal of PPCPs from water matrices has been associated with some challenges, among which are smaller particle size, generation of secondary pollutants, inability to reuse, or regenerate etc. Also, the use of semiconductors as catalysts in the degradation of PPCPs encounter the following drawbacks such as; requirement for high ultraviolet radiation for their activities due to wide bandgap energy within them; some are carcinogenic, e.g., TiO_2_; there is also the challenge with electron–hole pair recombination after use [8, 9]. Hence, the need to design materials that are green, economically efficient and effective methods for pollution control and prevention are needed for environmental protection and effluent discharge to have minimal impacts on human health and the biosphere.

Consequently, the advanced oxidation process (AOP) has been reported to be one of the most promising methods that can be employed for mineralization of complex organic pollutants as an effective alternative technique that involves the in-situ generation of strong oxidation radicals sufficient enough to degrade and eliminate organic pollutants within environmental matrices.

AOP is efficient in the removal of hazardous pollutants and their mineralization into non-toxic inorganic aliphatic acid, CO_2_ and water as compared to other decontamination techniques like; adsorption, coagulation, flocculation, sedimentation, bio-filtration, etc., as a result of the generation of active oxidizing agents such as hydroxyl radicals, superoxide, ozonide and photo-produced electron–hole pairs [10–12]. AOP can be achieved by direct ozonolysis, catalytic oxidation, homogenous/heterogeneous catalyzed oxidation, with the more recent advances in photocatalytic oxidation which is one of the green technologies that has attracted scientific interest as viable alternatives for the treatment of wastewater because of its low operating cost, nontoxicity, and effective reduction of contaminants [13, 14].

Over the years, efforts have been directed towards synthesizing various nanocomposites for photocatalysis purposes. This technique is one of the most preferred AOP because it offers the possibility of utilizing naturally available and renewable solar energy as a potential energy source containing visible light in the presence of a suitable photocatalyst prepared from metal oxides or semiconductors as catalyst immobilized on various solid supports for the photodegradation of pharmaceuticals. Unfortunately, the photocatalytic performance of most semiconductor photocatalyst is still low due to lack of enough active sites, large band gap, utilization of photons from the sun, and limited fast photoinduced charge carrier recombination [12, 15]. Hence, the development of efficient and highly stable photocatalysts with increased light absorption in the visible spectrum, reduced band gap, and decreased rapid electron–hole pairs recombination rate is necessary for the photocatalytic degradation of organic pollutants under visible light.

Hence, the aim of this review is to discuss the occurrence of pharmaceuticals and personal care products in the environment, highlights the failure of the conventional methods in degrading pharmaceuticals and personal care products from the water matrices and why photocatalytic degradation processes of pharmaceuticals and personal care products via the use of various semiconductor photocatalyst immobilized either on other semiconductors, metal oxide or on different carbon sources such as activated carbon, carbon nanotubes, and graphene oxides has been able to degrade these organic pollutants to propose more alternative nanomaterial that is green, efficient, non-toxic, cheap and more effective with a short time for the photocatalytic degradation of different organic pollutants.

## Pharmaceuticals and personal care products (PPCPs)

Pharmaceuticals and personal care products (PPCPs) consists of APIs, moisturizers, lipsticks, shampoos, hair colors, deodorants, toothpaste surfactant, and many household products that are mainly used for improving the quality of daily life [16] and their degradation products have been inadvertently present in the aquatic environment since the 1970s [17]. Their residues have been detected in all types of surface water, groundwater, and the oceanic environment in the last 20 years [18]. These compounds find their way into many aquatic environments receiving contaminated water from urban wastewater streams (domestic/industrial discharges, WWTPs effluents, etc.), which in turn get transferred into the water cycle, even reaching drinking water, as a result of their hydrophilic character and low removal efficiency of wastewater treatment plants (WWTPs).

Pharmaceuticals generally include prescription or over the counter (OTC) veterinary/human drugs and nutraceuticals administered for prophylaxis/therapeutic and health supplements purposes. They are classified as antibiotics, analgesics, blood lipid regulators, natural and synthetic hormones, β-blockers, anti-diabetics, antihypertensive, and many more products that are used for health purposes [19]. These substances are continuously discharged into the aquatic environment from a point and non-point domestic and industrial sources.

## Presence and occurrence of pharmaceuticals and personal care products in wastewater

A global survey has indicated the presence of several classes of PPCPs in most aquatic environments. Unfortunately, these data rely primarily on studies conducted in Europe, North America, Scandinavia, and a few other places. The availability of such data in those parts of the world is because research on PPCPs and many endocrine disruption contaminants/pollutants in African Countries and especially in the South Africa aquatic system is scanty [20, 21]. Despite the lack of data, the occurrence of some PPCPs at concentration levels that sometimes surpassed µg/L level in WWTPs effluents and environmental waters have been reported [22–24]. Research studies on remediation of contaminated/polluted aqueous matrices and or the removal of these contaminants in drinking treatment plants and or during wastewater treatment before discharge to surface water is still far behind and probably lacking.

Commonly detected pharmaceuticals in different environmental matrices and their occurrence levels are as listed in Table 1 below.

**Table 1:** Commonly detected PPCPs in different environmental matrices and their occurrence levels around the world

PPCPs	Concentration (µg/L)	Country	Source	References
Sulfamethoxazole	0.33–0.61	Beijing	River and sewage water	[25]
	2.00	Australia	Surface water	[26]
	3.00	Australia	Wastewater treatment plant influent	[26]
	0.20	Australia	Wastewater treatment plant effluent	[26]
	34.50	Kwazulu-Natal, South Africa	Wastewater treatment plant influent	[24]
	3.68	Kwazulu-Natal, South Africa	Surface water	[22]
	0.049	Scaynes Hill, UK	Wastewater treatment plant influent	[27]
	0.023	Scaynes Hill, UK	Wastewater treatment plant effluent	[27]
Nalidixic acid	1.73–30.84	Kwazulu-Natal, South Africa	Surface water	[22]
	29.9	Kwazulu-Natal, South Africa	WWTW influent	[28]
	25.2	Kwazulu-Natal, South Africa	WWTW effluent	[28]
	0.20	Australia	WWTW influent	[26]
	0.45	Australia	WWTW effluent	[26]
	0.75	Australia	Hospital effluent; Wastewater treatment plant	[26]
Aspirin	0.664	South Wales, UK	Cilfynydd wastewater treatment influent	[29]
	0.027	South Wales, UK	Cilfynydd wastewater treatment effluent	[29]
	2.49	South Wales, UK	Coslech Wastewater treatment Influent	[29]
	0.02	South Wales, UK	Coslech Wastewater treatment effluent	[29]
	2.566	Catalonia, Spain	Wastewater Influent	[30]
	0.034	Catalonia, Spain	Wastewater effluent	[30]
	0.012	Catalonia, Spain	Surface Water	[30]
	874	Canada	Influent sewage treatment plants	[31]
	59.6	Canada	Effluent sewage treatment plants	[31]
	118.00	Kwazulu-Natal, South Africa	Wastewater treatment plant influent	[28]
	44.20	South Africa	Wastewater treatment plant effluent	[28]
Naproxen	0.838	South Wales, UK	Cilfynydd wastewater treatment influent	[29]
	0.37	South Wales, UK	Cilfynydd wastewater treatment effluent	[29]
	1.173	South Wales, UK	Coslech wastewater treatment influent	[29]
	0.17	South Wales, UK	Coslech wastewater treatment effluent	[29]
	5.938	Korea	WWTP influent	[32]
	0.120	Korea	WWTP effluent	[32]
	52.30–55.00	Gauteng, South Africa	WWTP influent	[23]
	13.50–20.40	Gauteng, South Africa	WWTP effluent	[23]
Ibuprofen	1.68	South Wales, UK	Cilfynydd wastewater treatment influent	[29]
	0.26	South Wales, UK	Cilfynydd wastewater treatment effluent	[29]
	2.29	South Wales, UK	Coslech wastewater treatment influent	[29]
	0.14	South Wales, UK	Coslech wastewater treatment effluent	[29]
	5–8	Canada	Wastewater effluent	[33]
	62.82	Kwazulu-Natal, South Africa	WWTW influent	[24]
	58.71	Kwazulu-Natal, South Africa	WWTW effluent	[24]
	9.494	Korea	WWTP influent	[32]
	0.015	Korea	WWTP effluent	[32]
Acetaminophen	211.38	South Wales, UK	Cilfynydd wastewater treatment influent	[29]
	11.73	South Wales, UK	Cilfynydd wastewater treatment effluent	[29]
	178.12	South Wales, UK	Coslech wastewater treatment influent	[29]
	0.35	South Wales, UK	Coslech wastewater treatment effluent	[29]
	11.3	France	WWTP influents	[34]
	5.76	Kwazulu-Natal, South Africa	WWTW influent	[24]
	74.552	Korea	WWTP influent	[32]
	0.023	Korea	WWTP effluent	[32]
Carbamazepine	1.69	South Wales, UK	Cilfynydd wastewater treatment influent	[29]
	2.49	South Wales, UK	Cilfynydd wastewater treatment effluent	[29]
	0.95	South Wales, UK	Coslech wastewater treatment influent	[29]
	0.83	South Wales, UK	Coslech wastewater treatment effluent	[29]
	1.9	Canada	Wastewater treatment influent	[31]
	2.3	Canada	Wastewater treatment influent	[31]
	0.42	Tainan, Taiwan	WWTP effluent	[35]
Diclofenac	22.3	Kwazulu-Natal, South Africa	Wastewater treatment plant influent	[28]
	12.4	Kwazulu-Natal, South Africa	Wastewater treatment plant effluent	[28]
	15.3–19.4	Germany	Wastewater treatment plant	[19]
	0.21–0.49	France	WWTP effluents	[34]
	0.397	Scaynes Hill, UK	Wastewater treatment plant influent	[27]
	0.119	Scaynes Hill, UK	Wastewater treatment plant effluent	[27]
	0.251	Sapporo, Japan	Municipal wastewater treatment plant influent	[36]
	0.145	Sapporo, Japan	Municipal wastewater treatment plant effluent	[36]
Tetracyclines	0.10	Australia	WWTW influent	[26]
	0.02	Australia	WWTW effluent	[26]
	0.6–5.7	Kwazulu-Natal, South Africa	Surface water	[22]
	0.003–0.008	Tianjin China	Municipal wastewaters influents	[37]
Bisphenol A	1.52	Tianjin China	Municipal wastewaters influents	[37]
	0.190–1.0789	Harbin city, China	Wastewater influents	[38]
	0.0614–0.1755	Harbin city, China	Wastewater effluent	[38]
	0.028	Pretoria and Cape Town, South Africa	Drinking water	[39]
	0.002	Germany	Drinking water	[40]
	0.047	Germany	Sewage Treatment effluents	[40]
	0.014	Germany	River Water	[40]
Triclosan	78.40–127.70	Gauteng, South Africa	WWTP influent	[23]
Benzophenone	1.5–8.58	Nagpur city, India	Sewage treatment plant	[41]
	10.70–22.90	Gauteng, South Africa	WWTP effluent	[23]
Estrone	0013–0.351	Kwazulu-Natal, South Africa	WWTW influent	[42]
	0.078–0.158	Tianjin China	Municipal wastewaters influents	[37]
	0.003–0.078	Kwazulu-Natal, South Africa	WWTW effluent	[42]
	0.0102–0.0349	Harbin city, China	Wastewater influents	[38]
	0.0083–0.014	Harbin city, China	Wastewater effluents	[38]
17-β-Estradiol	0.02–0.199	Kwazulu-Natal, South Africa	WWTW influent	[42]
	0.004–0.107	Kwazulu-Natal, South Africa	WWTW effluent	[42]
	0.011–0.054	Tianjin China	Municipal wastewaters influents	[37]
	0.0466–0.093	Harbin city, China	Wastewater influents	[38]
	0.0087–0.0324	Harbin city, China	Wastewater effluents	[38]
	0.0041	California, USA	Municipal wastewater effluent	[43]
Estriol	0.003–0.009	Kwazulu-Natal, South Africa	WWTW influent	[42]
	0.042–0.162	Tianjin China	Municipal wastewaters influents	[37]
Progesterone	0.163–0.904	Kwazulu-Natal, South Africa	WWTW influent	[42]
	0.025	Kwazulu-Natal, South Africa	WWTW effluent	[42]
Testosterone	0.119–0.635	Kwazulu-Natal, South Africa	WWTW influent	[42]
	0.026	Kwazulu-Natal, South Africa	WWTW influent	[42]

## Classification of pharmaceuticals and personal care products

Pharmaceuticals can be classified into different active organic groups of compounds [44, 45]. These include but not limited to; (i) antibiotics which comprise penicillin, tetracycline, sulfonamides, macrolides, fluoroquinolones, and β lactams; (ii) steroids hormones many of which have been implicated as endocrine-disrupting compounds such as estrogens, estrone, estriol, 17-β-estradiol, 17-α-ethinylestradiol, testosterone, etc. (iii) analgesic and non-steroidal anti-inflammatory drugs (NSAIDS) which are one of the most prescribed groups of pharmaceuticals that include acetaminophen, diclofenac, ibuprofen, and naproxen; (iv) antiepileptic drugs such as carbamazepine, used in reducing the frequency of epileptic seizures; (v) blood lipid regulators, e.g., bezafibrate, gemfibrozil, fenofibric acid and clofibric acid; (vi) β-blockers such as salbutamol, atenolol, sotalol, theophylline and metoprolol used in treating hypertension and cardiac dysfunctions; (vii) antineoplastic such as cytostatic drugs used in cancer therapy; etc. Antimicrobial agents, fungicides, disinfectants, synthetic musks, some preservatives, some sunscreen UV filters, etc., may also be referred to as pharmaceuticals depending on the description of use.

**Fig. 1:**
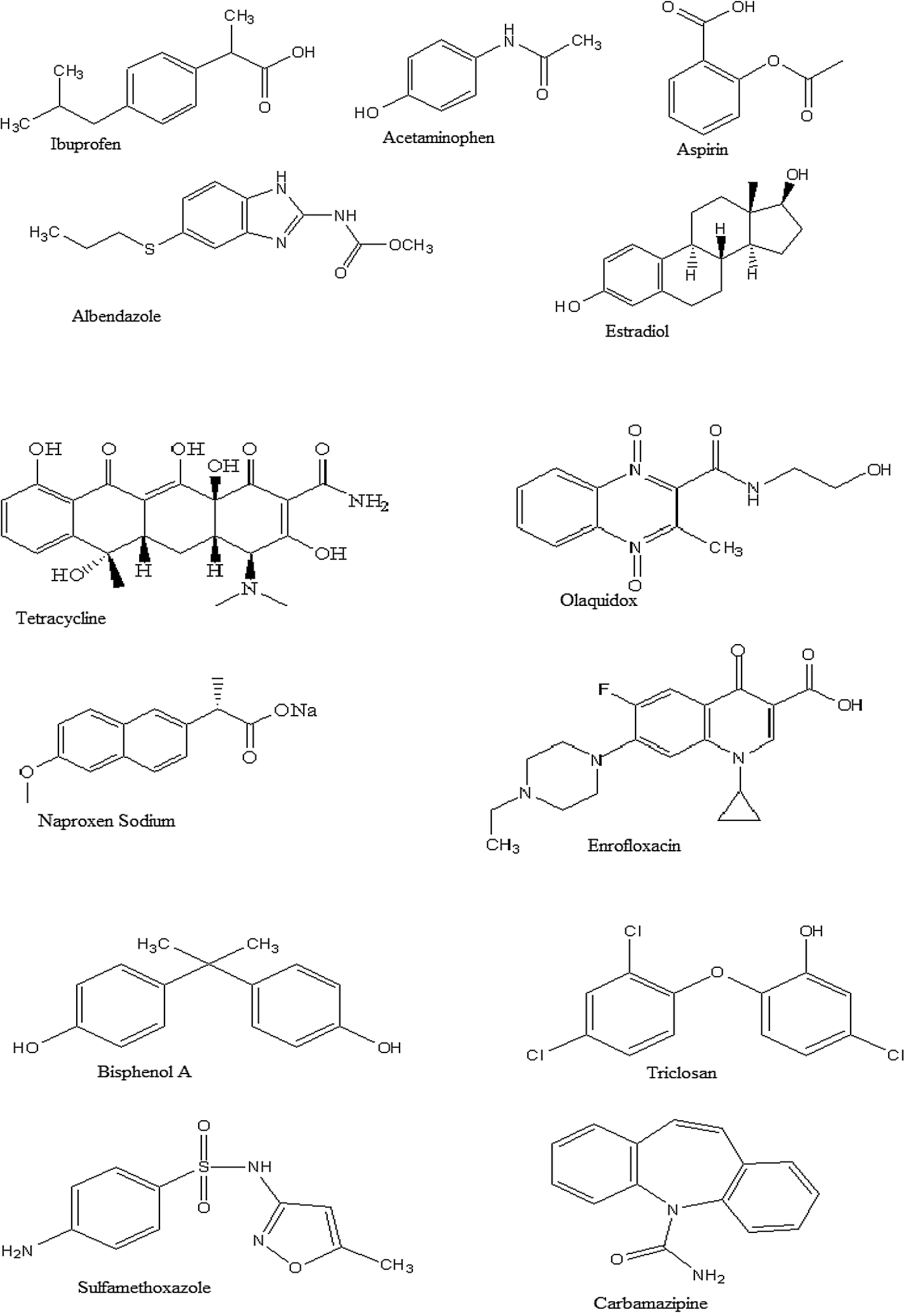
Structures of some pharmaceuticals and personal care products

Personal Care Products (PCPs) groups include; (i) pesticides/insect repellants, (ii) musk; widely used as fragrances of many personal care products such as; perfume, body lotion, hair care products, shower bath products facial essence and numerous household products, (iii) soaps and detergents, (iv) sunscreen UV filters, which are chemicals that serve as absorbers and protection of the skin from different ultraviolet radiations from UV-induced damages, (v) triclosan; a lipid-soluble, broad-spectrum, antimicrobial agents which are used as preservatives in personal care products such as hand soap, shampoos, detergents, toothpaste, sunscreen, deodorants, and (vi) antiseptic which serves various purposes in medical devices, household items and as additives in packaging textiles and functional clothing [46–48]. Some of their structures are given in Fig. 1

## Source of PPCPs in the environment

The global annual consumption of PPCPs in developed world and countries such as Brazil, Russia, India, China, and South Africa has been reported to have increased over the last decade, due to their medical use for prophylaxis/therapy, and economic use for viability sustenance of commercial aquaculture and livestock agriculture [49]. As a result, residues of compounds of PPCPs are continuously released into wastewater via wash-off, urine, and feces as parent compounds, derivative conjugates, or metabolites [50]. PPCPs can also enter the surface water through direct discharge from industries [51], hospitals, household’s/domestic wastewater and through surface runoff (as result of the use of contaminated biosolids as manure spread on agricultural land), reaching water bodies and groundwater by leaching or bank filtration. Other means through which PPCPs enter into the aquatic environment include disposal of unused medicines into landfills, irrigation with wastewater, off-label emissions, and disposal of carcasses of treated animals [52], and sewage or wastewater treatment plants [53, 54]. Brauschet al. [55] reported that the WWTPs are the main route through which PPCPs are discharged into the aquatic environment. They have been found in lakes, rivers, groundwater, marine, and coastal area waters and drinking water [45, 56, 57].

**Fig. 2:**
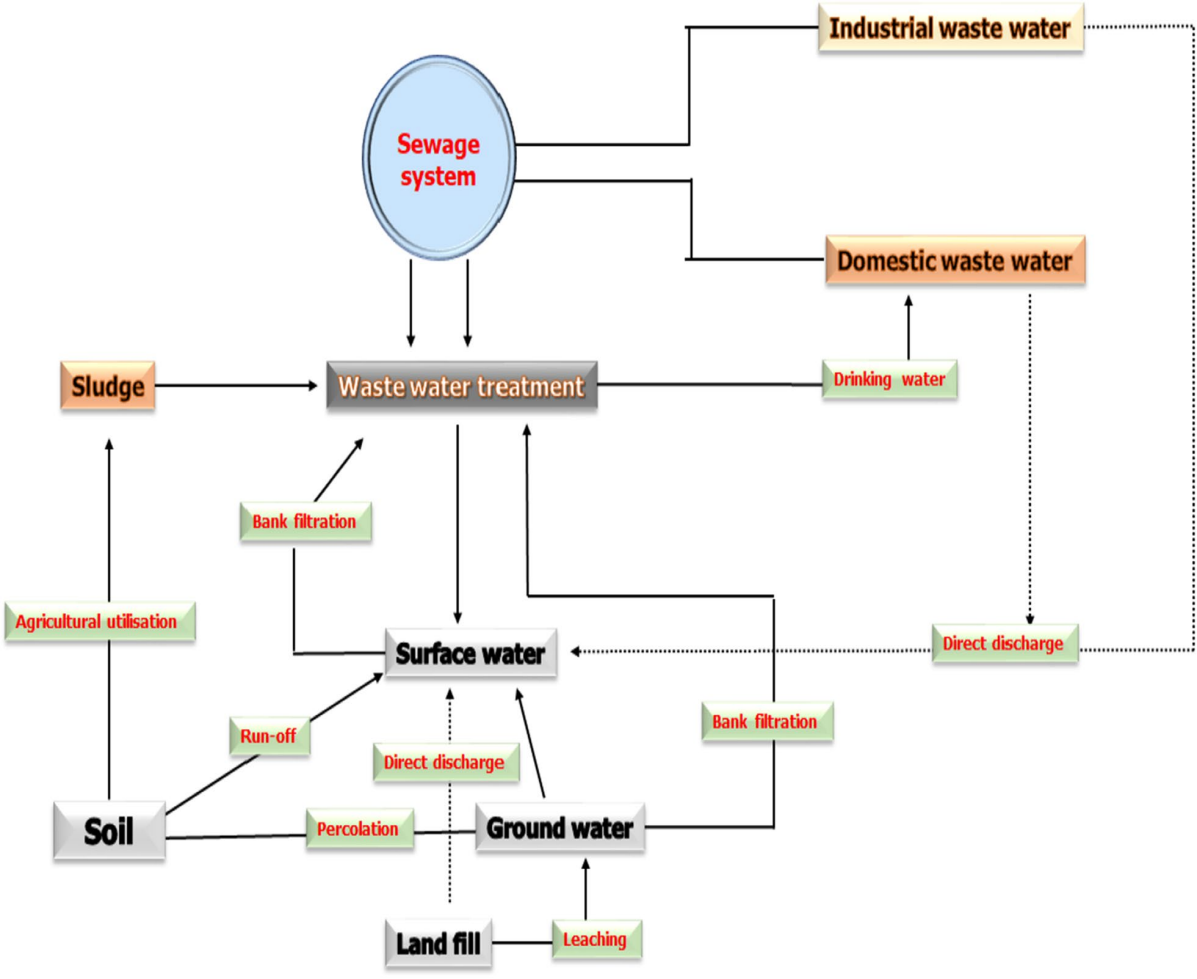
Schematic diagram showing potential sources, pathways, and receptors of pharmaceutical pollution in the environment (adapted with modification from [60])

Aquatic sediments can also hold a substantial amount of PPCPs because of their affinity binding/sorption capacity [58]. Thus, excess PPCPs do not remain in the water for long, as they partition between water, sediment, and other components of the aquatic ecosystem, via diffusion, seeding, silting out of suspended particle bonded-PPCPs and other physical/chemical processes. Aside from the application of contaminated biosolids on farmland soils, several PPCPs reach the soil and different terrestrial domain through the deposition (air/wet) of short and long-range atmospheric transferred aerodynamic sized PPCPs particulates [59]. A schematic diagram showing potential sources and pathways of pharmaceuticals is shown in Fig. 2.

## Transformation of PPCPS and their metabolites

The soluble characteristics and bioactive nature of many pharmaceuticals and some personal care products may facilitate their biotransformation and depuration in biological features. However, this is not the case with most PPCPs in the environment, where they do not readily degrade. Many pharmaceuticals are discharged from the human and animal bodies in a mixture of their parent compound and conjugate metabolites [61, 62]. The extent of body metabolism and pharmacokinetics index is a function of the ratio between the parent compound (un-metabolized) and the conjugate metabolites concentrations measured in urine or feces [63, 64]. They may also undergo environmental transformation under certain conditions (enviro-kinetic).

Monitoring metabolites of pharmaceuticals in the environment is, however, limited by the lack of reference standards of environment bound pharmaceutical metabolites, resulting in the scanty information on the occurrence levels, behavior, and half-life of conjugates metabolites in the environment. For instance, Kasprzyk-Hordern et al. [29] and Huerta-Fontela et al. [65] reported a concentration range of between 880 and 4026 ng/L for carbamazepine epoxide (a carbamazepine metabolite) in influents wastewater, while the concentration of its parent compounds (carbamazepine) range between < 1.5 and 113 ng/L. There is, therefore, a need to understand environmental transformation/metabolism, transformation products (metabolites), and analysis of pharmaceutical metabolites. This action is essential because of the expected time-bound decrease in the environmental concentration of parent compounds, with a corresponding increase in metabolites’ level, as well as the potential of the metabolites, eliciting probably greater than parent compound adverse effects in the environment.

## Toxicity of pharmaceuticals and personal care products

The benefits of the intended use of pharmaceuticals and many personal care products are enormous. The consumption of drugs evokes specific biological responses in the host, after which 10–90% of excess unused doses and some conjugates metabolites are ultimately excreted and passed into the environment [66, 67]. Their leak and presence in different environmental matrices could cause a risk on non-target organisms, mainly because of their weak degradation, persistence, and biological activities. Organisms within the aquatic and terrestrial environments have the potential to be most affected. Recent reports indicated that drinking water sources, especially those receiving water input from contaminated sources, contain residues of PPCPs, and thus may need further treatment to remove the pollutants before consumers supply [68].

There has been a growing concern about the environmental impact of pharmaceuticals and personal care products (PPCPs) in the aquatic environment [69] as a result of the fact that they can potentially elicit health and ecological impacts [55]. Furthermore, PPCPs may exist as mixtures with other contaminants in the environment and, therefore, can cause synergistic detrimental effects to both aquatic and terrestrial organisms even at low concentrations [70]. Antibiotics usage is common in animal husbandry for prevention, treatment, and growth purposes, making their over-use result in the genetic selection of more harmful bacteria [71]. Continuous exposure to antibiotics was noted as a probable cause of induced drug resistance in many pathogenic organisms, including bacteria.

Steroid hormones use as sex steroids, contraceptives, and birth control pills can interfere with the endocrine systems, or act as anti-androgenic ligands [72, 73]. Endocrine disruption leading to adverse effects such as sterilization, the imposition of sex organs (imposex), and feminization of some vertebrate, e.g., fish, mollusks, and other aquatic species have been reported [5, 74]. For instance, Solé et al. [75] reported that the presence of the hormone estrogen and progestogens at concentrations as low as approximately 1.0 µg/L caused endocrine-disrupting manifesting in fish feminization and decrease infertility. The accumulation of diclofenac on *rainbow trout* even at a low concentration of 5 µg/L had detrimental endocrine effects. In contrast, exposure to a mixture of acetaminophen, carbamazepine, gemfibrozil, and venlafaxine at concentrations between 0.5 and 10 µg/L was noted to cause effects such as tissue degeneration, a decline in embryo production and increase in embryo mortalities in *zebrafish* [76, 77]. Exposure to endocrine-disrupting contaminants in the aquatic environment was also reported to lead to the modulation of hypothalamus–pituitary–gonad (HPG), hypothalamus–pituitary–thyroid (HPT) and hypothalamus–pituitary–adrenal (HPA), and resulting in the tampering of the activities and functions of various physiological traits in some non-target vertebrate aquatic organisms [78].

Drugs such as carbamazepine, levetiracetam, lamotrigine, and valproate used as antiepilepsy which have been detected in drinking water supply in South Africa was fingered to have the capacity to cause a number of several reproductive endocrine system side-effects in certain fish species as well as in humans [77, 79, 80]. NSAIDs like Naproxen and Ibuprofen that are often not resolved in WWTPs may also induce an adverse effect on the endocrine systems of non-target vertebrate organisms of WWTPs effluent receiving waters. For example, Japanese Medaka fish (*Oryzias latipes*) exposed to 0.1 µg/L concentration of Ibuprofen manifested delayed hatching while concentration reaching 1 mg/L in humans, can lead to an increase in blood plasma levels.

## Remediation, treatment, and removal of PPCPS from contaminated matrices

Extraneous levels of widely distributed potentially toxics PPCPs and EDCs have been reported in many aqueous matrices worldwide. A large percentage of South Africa waters and aquatic ecosystem receives PPCPs contaminants via human excretions and improper discharge of pharmaceuticals into the sewage system [42, 81, 82]. Consequently, many non-target marine organisms have magnified drug residues to a varying extent in different organs of their bodies. PPCPs such as acetaminophen, caffeine, 1,7-dimethylxanthine, dehydronifedipine, tetracycline, oxytetracycline, sulfonamides, macrolides, and ormetoprim have been reported to be found in fruits, vegetables, fish, meat and even milk and dairy products as a result of uptake or exposure to PPCPs contaminated environment [83–85]. Thus, there is a need for the implementation of appropriate and effective environmental remediation to secure the health and safety of biological features, as well as environmental health and safety.

Several water treatment procedures and technologies have been developed and used for resolving contaminants in the environment, especially water and wastewater. The removal can be achieved through several processes, including; physical, biological, and chemical methods. The physical process may involve filtration where contaminants’ removal is achieved by transforming one phase to another, leading to the production of highly concentrated sludge that could be toxic and more difficult to dispose of [86]. The biological process in water treatment revolves around aerobic and anaerobic degradation/digestion of nutrients and organics, while the chemical process is based on oxidative decomposition. However, many of these stepwise treatment techniques are insufficient, in that they are not able to remove or even degrade residues of PPCPs due to their deficient environmental concentrations, and because they were not initially factored in their design for such purposes [87]. Daughton and Ternes [44] reported that PPCPs are not entirely removed during wastewater treatment because the WWTPs are only designed to remove suspended solids, pathogens, and nutrients from wastewater using a serial link of processes to eliminate impurities, reduce nutrients and organic load and improve oxygen demand levels.

Petrović et al. [88], in a report, noted that the majority of the existing WWTPs are not designed to either treat or remove these contaminants and their metabolites. Ineffective treatment of wastewater has been listed as a significant source of PPCPs in many receiving fresh surface water systems and other aquatic environments, especially those freshwater systems used as domestic water sources. There is, therefore, a need for the development of more advanced, efficient, and powerful water and wastewater treatment technologies for the treatment of PPCPs contaminated water, wastewater/effluent from municipal and industrial wastewater discharge streams [89]. This measure is critical and very important in eliminating the potential toxicity and health implication that may arise as a result of exposure of aquatic organisms and possibly humans to these metabolites. The development of facile, cheap, and green methods that can be employed in the treatment of pollutants has been a significant area of research in the field of environmental science and technology. It is important to note that the ability of certain PPCPs to sorb onto bio-solids and siliceous solids, suspended solids confers the need for remediation of contaminated sediment as well as contaminated soil, where they may be ultimately sunk into, or be discharged onto via the application of bio-solids as manure on land.

## Water treatment and removal efficiency of PPCPs in WWTPs

Over 80% of PPCPs are released into the environment from the human body in the parent form un-metabolized, i.e., without transformation [90]. These are fed through into the wastewater treatment plants for removal or decomposition during the water treatment process. Many WWTPs are, however, unable to wholly eliminate most PPCPs, hormones and their metabolites, resulting in their presence as residues in wastewaters, surface waters, sediments, and sludge. This inefficiency of WWTPs could be attributed to their conventional integrated treatment uses for biological treatment and microbial decomposition as a process for the breakdown of complex organics, nutrients, and activated sludge for the removal method of contaminants in water/wastewater. Unfortunately, residues of PPCPs, hormones, and their metabolites are not fully removed as the removal efficiency of most WWTPs ranged between 50 and 90% [91, 92]. The removal efficiency of different PPCPs differs greatly, and varies with the physical and chemical properties of the parent compounds as well as the environmental conditions such as the configuration of biological reactors and operational parameters such as hydraulic retention time and sludge retention time [93, 94].

For example, carbamazepine, one of the contaminants that are most often detected in effluents, is known to be resistant to biodegradation, making it nearly impossible for efficient mineralization and removal in conventional biological treatment [20]. Wu et al. [95] reported that 23% removal efficiency for carbamazepine after treatment of wastewater effluent in the conventional treatment process. The removal efficiency of other PPCPs such as ibuprofen, naproxen, ketoprofen, diclofenac, bezafibrate, sulfamethoxazole, and trimethoprim ranges from 60 to 100% [96–99]. The differences in the removal efficiency may be due to a number of factors, including the characteristic of the activated sludge, the concentration of the contaminants, the optimal operational condition of the WWTPs, and the water composition. Thus, the biological treatment process seems to be economically feasible and widely applicable but not effective in degrading many organic pollutants.

Most WWTPs utilizes various method in the conventional treatment systems for the removal of PPCPs such as coagulation, flocculation, and sedimentation of raw sewage, activated sludge, membrane bioreactor, sequential batch reactor up-flow anaerobic sludge blanket processes [100, 101]. Some treatment methods are, however, more efficient and effective in removing pharmaceuticals compounds of interest, compared to another. This might be as a result of the physicochemical properties, the hydrophobicity, and biodegradability of target pharmaceuticals and process operating parameters in the WWTPs [102]. For instance, Berset et al. [103] reported that using the conventional methods involving dioxychlorination and sand filtration processes, cocaine and benzoylecgonine were poorly removed, while they were removed entirely when ozonation process was used. Non-steroidal anti-inflammatory drug removal in WWTPs are achieved to a different extent. Upon treatment with a mixture of dioxychlorination and sand filtration, diclofenac (over 99%) was almost completely removed; naproxen was moderately removed (48%), while ibuprofen was poorly removed (14%). When ozonation process was used as a treatment process, more than 40% ibuprofen, naproxen, and diclofenac were removed by less than 20%, while dioxychlorination and sand filtration process remove sulfonamides and macrolides efficiently [104]. Thermal destruction of pollutants at high temperatures is also a suggested elimination procedure. However, this technique may lead to undesirable emissions and damage to useful microbes and aquatic life forms, while it is not economically feasible.

## Biosorptive method for the removal of PPCPs from contaminated water

Wastewater treatment plants (WWTPs) rely on biodegradation and adsorption methods using conventional adsorbents such as activated sludge for removal of contaminating substances in aqueous matrices, but their use is limited. This limitation is because their removal efficiency changes significantly, and varies with the physicochemical properties of the compounds as well as the environmental conditions and operational parameters [58, 105]. The biological, chemical, and physical processes have been studied for the removal of PPCPs from contaminated matrices [106]. Among these techniques, biosorptive method is seen as one of the environmentally friendly techniques used in the removal of PPCPs from aqueous solutions. Bioadsorptive immobilization have been reported to show promising results for the removal of some organic contaminants in laboratory pilot-scale experiments while biodegradation was proven to be inefficient for the removal of PPCPs due to poor degradability and low environmental concentration [107, 108].

Biosorptive processes have the advantages of effectiveness, simplicity, low cost, and sustainability [58]. Despite these advantages, the efficiency of bio-adsorption varies depending on the characteristics, polarity, the activity of the sorbent materials, the chemical properties and behaviours of the adsorbate material, thus suggesting incomplete pollutant removal and thereby passing them out in WWTP effluent. The cost of WWTP operation due to high energy requirements and low output is also a limitation [89]. There is also a tendency of the transformation of some non-biodegradable contaminants into toxic contaminants that can exert toxicity to micro-organisms under the treatment conditions [109].

Commercial activated carbon as absorbents is one of the most widely studied materials for the removal of organic pollutants. Still, its drawback is in the high manufacturing cost and the deficiency of regeneration. Hence the preparation of economical adsorbents from agricultural waste, clay materials, biomass, non-living algae, and seafood-processing waste products, which are cheap, have been employed as biosorptive material for the removal of numerous organic pollutants from different water matrices [110]. Li et al. [111] reported the high removal efficiencies of diclofenac and trimethoprim via the use of agro-waste materials such as biochar, macro-algae, and wood chipping. These biosorptive materials indicated possible high removal at low concentrations, thereby offering a clean, sustainable, and practical water treatment solution for purifying water.

Alkaline-modified biomass employed for the removal of tramadol achieved a 91% removal at pH of 7, biosorbent dose of 0.5 g/L, and 50 mg/L contaminant concentration load of tramadol within 45 min. The removal efficiency of the sorbent was possible because of the hydrophilic interaction between amino and carbonyl groups within the pharmaceuticals moieties, and the hydroxyl and carbonyl functional groups generated on the surface of the modified algal biomass [110]. Moreover, different biosorbents such as graphene oxide and carbon nanotubes have been used in the removal of various pharmaceuticals and personal care products [112–114]. Solution pH, temperature, interfering substances, ionic strengths of the matrix are among the environmental factors that play an essential role during biosorptive process for the removal of PPCP. However, their inability to be able to remove a large number of pharmaceuticals from wastewater treatment plants efficiently calls for the development of an efficient technology with acceptable cost and effective for mineralization of pharmaceuticals from the wastewater treatment plants.

## Chemical oxidation methods for the dissipation and mineralization of PPCPs in water

Chemical oxidation method offers a better technology in that the challenges associated with the use of conventional wastewater treatment plants, which include incomplete degradation of organic pollutants, sludge formation, the formation of secondary pollutants, and high cost, are eliminated. Chemical oxidation generally overcomes the limitations of other treatment processes, although it may not result in the complete mineralization of the organic pollutants [115]. Chemical oxidation, often referred to as an AOP, involves the use of oxidants like ozone, hydrogen peroxide, and chlorine.

Chemical treatment method involving advanced oxidation processes such as ozonation, peroxidation, ultraviolet light aided ozonation (O_3_/UV), ultraviolet light aided peroxidation (UV/H_2_O_2_), Fenton and Fenton-like oxidation, gamma radiolysis, sonolysis as well as electrochemical oxidation process have been reported to be sufficient for the degradation of toxic pollutants in the aqueous matrices [116]. Howbeit, the advanced oxidation process methods have been associated with the disadvantage of high energy demand for various critical devices such as ozonizers, UV lamps, and ultrasonicators, and this leads to high operation costs [117].

Basically, advanced oxidation processes rely on in situ generation of highly potent chemical oxidants in the presence of ozone (O_3_) or hydrogen peroxide (H_2_O_2_) and the presence of a catalyst such as the Fenton’s reagent or UV light [118]. AOPs uses reactive species such as the hydroxyl radicals OH· generated in sufficient quantity for water purification, in which both organic and inorganic pollutants are removed through O_3_ aided oxidation or H_2_O_2_ supported oxidation, in the presence of catalysts such as TiO_2_, ZnO, BiO_2_, CuO etc., and UV light as an energy source at ambient temperature and pressure [119]. AOPs are classified into two based on photon-driven reactions;

- Photochemical processes such as UV oxidation, UV/H_2_O_2_, UV/O_3_, UV/H_2_O_2_/O_3_, UV/Ultrasound, Photo-Fenton, photocatalysis, sonophotocatalysis, vacuum UV, microwave, and,
- Non-photochemical processes such as ozonation, ultrasound, US/H_2_O_2_, US/O_3_, US/Fenton, electrochemical oxidation, supercritical water oxidation, ionization radiation, electron-beam irradiation, wet-air oxidation [120].

AOPs excel overall biological and chemical processes in that they are environmentally friendly as they are known not to transfer pollutants from one phase to the other, as seen during chemical precipitation, adsorption, and volatilization [120]. Moreover, they do not produce a large quantity of poisonous sludge during activated sludge processes as with other treatment methods. Popular advanced oxidation processes used for the removal of contaminants include TiO_2_ and photo-fenton processes. Generally, photocatalyzed AOP is environmentally benign, biocompatible, highly stable with low-cost metal oxide photocatalyst (such as TiO_2_), and can efficiently be used to remove different organic pollutants from water matrices [121, 122]. The photocatalytic degradation of organic pollutants within the water matrices are very efficient and effective due to the following factors; (i) good operating conditions at ambient temperature and pressure, (ii) complete mineralization of substrate and intermediate into CO_2_ and H_2_O, (iii) no problem with disposing of solid waste, (iv) utilization of sunlight, near UV-light or visible light for irradiation, (v) formation of non-toxic by-products and (vi) low cost [12, 123].

TiO_2_ photocatalysis, however, offers better activity during the removal of organic pollutants because of their heterogeneous nature, hence, the possibility of catalyst reuse and their operation at a wide pH range when compared to photo-fenton process. Although different AOPs method has been employed in the degradation of PPCPs, however, this review intends to emphasize on the catalytic activity of semiconducting photocatalyst doped on solid supports such as activated carbon, carbon nanotubes, and graphene oxides and their application in the degradation of PPCPs. One of such semiconducting photocatalyst is TiO_2,_ or TiO_2_ immobilized on other solid materials used in AOPs processes. Photocatalysis occurs upon the absorption of ultraviolet, visible, or infrared radiation by the photocatalyst in such a way that results in a change in the rate of the chemical reaction. The use of catalysts such as TiO_2_ or TiO_2_ immobilized on a support material was reported to lead to improved oxidation under solar irradiation, while the radiation also serves as a more sustained and energy-efficient solution for degradation of organic contaminants in the aqueous environment [124, 125].

Photocatalysis can either be a homogenous or heterogeneous photocatalyst. Homogenous photocatalysis occurs when both the photocatalyst and the organic pollutants are soluble and exist in the same phase during the photocatalytic process. An example of homogenous photocatalyzed AOP is the photo-Fenton process, which involves the iron salt catalyzed H_2_O_2_ reaction in the presence of UV–visible radiation as an energy source used for the generation of hydroxyl radicals as shown in the reaction below [126].

1 $$ {\text{Fe}}^{2 + } + {\text{H}}_{2} {\text{O}}_{2} \to {\text{Fe}}^{3 + } + {\text{OH}}^{ - } + {\text{OH}}^{ \cdot } $$

2 $$ {\text{Fe}}^{3 + } + {\text{H}}_{2} {\text{O}}_{2} \to {\text{Fe}}^{2 + } + {\text{HO}}^{{2{\mathbf{ \cdot }}}} + {\text{H}}^{ + } $$

3 $$ {\text{Fe}}^{3 + } + {\text{HO}}^{{2{\mathbf{ \cdot }}}} \to {\text{Fe}}^{2 + } + {\text{O}}_{2} + {\text{H}}^{ + } $$

4 $$ {\text{Fe}}^{3 + } + {\text{H}}_{2} {\text{O}} + h\upsilon \to {\text{Fe}}^{2 + } + {\text{H}}^{ + } + {\text{OH}}^{ \cdot } $$

This process is used in the degradation of pharmaceuticals as a result of its simplicity and easy reactor design. Photo-Fenton processes have been applied for the degradation of organics pollutants in different environmental matrices. de Luna et al. [127] reported the degradation of acetaminophen by Fenton oxidation in a fluidized-bed reactor where the effect of operating parameters such as pH, temperature, the concentration of H_2_O_2_ and Fe^2+^ were monitored for the pseudo-second-order kinetic degradation 99.6% of the organic substrate. The Fenton process has also been proven to be a viable catalyst for photo-degradation of pharmaceuticals such as hydrocortisone, estradiol, and verapamil. The reaction mixture containing ferric ions as catalyst showed 100% degradation of the three pharmaceuticals in less than 1 h, compared to the use of TiO_2_ in a heterogeneous photocatalyzed process which shows a more favorable level of mineralization which in turn prevent the danger involved in the accumulation of harmful organic pollutants in the water body [128].

The drawbacks in the use of this process include the use of reagents, strict pH control due to the process’s pH dependence, and the formation of sludge. To overcome these challenges, the development of heterogeneous catalysts for the oxidation degradation processes was crucial, due to their potential role in facilitating the degradation and complete mineralization of organic contaminants, especially PPCPs. Current heterogeneous catalyst development studies are directed towards improving the synthesis, and functionality of various sizes and shapes of metal nanoparticles semiconducting photocatalyst, aimed at improving the performance and utilization of nanoparticles in multiple applications, including advanced oxidation processes (AOPs). In general, the use of ozonolysis and photocatalytic oxidation has proven to be promising as pre-treatment/treatment techniques for the mineralization of organic pollutants when compared with other chemical oxidation processes.

### Ozonolysis processes

The ozonolysis process is an advanced oxidation process that is based on the synergistic interaction between an oxidant such as ozone or abundant oxygen. Ozonolysis may be supported by catalyst or radiation or a combination of both resulting in the generation of hydroxyl radicals (HO·), which is a short-lived oxidizing species that reacts with both organic and inorganic pollutants within the water matrices in a non-selective manner [129]. Due to AOPs ability for selective degradation of recalcitrant organic pollutants, they may function as a pretreatment process prior to biological treatment techniques, thus suggesting integrated treatment as a more efficient and effective means for the removal of organic pollutants from highly contaminated wastewater [130, 131].

The preference for the use of ozonolysis in the removal of pollutants is because they do not cause a significant increase in salt concentration. It leaves no presence of chemical residue after usage, as observed with waste activated sludge, where bio-recalcitrant components and low soluble volatile organic compounds, are partially oxidized and solubilized to produce a biodegradable product [132, 133]. However, this method is expensive and requires a high operating cost, thereby making its application for pollutants removal limited. The technique is also associated with the generation of intermediates, which could be challenging to eliminate.

### AOP using heterogeneous photocatalysts

This process is made up of purely chemical and physical reaction routes in which the photocatalyst is generated and its activity maintained by the absorption of radiant energy [134]. Heterogeneous photocatalysis occurs when the activity of semiconducting photocatalyst, which in most cases is insoluble in the solution that contains the organic substrates, and dependent on the pH of the solution, is initiated by the absorption of radiant energy. The generated photon with energy band gap level equal to or greater than the band gap energy of the photocatalyst which is the difference between the energy of the valence band full of electrons and that of the conduction band that is empty. Then, mobile electrons can access the conduction band, leaving positive holes in the valence band [131, 135]. Hence the catalyst and the substrates exist in different phases. The heterogeneous nature of the oxidative reaction makes for easy and cheap recovery catalyst from the solution after use.

The conventional semiconducting nanoparticles used in AOPs for the treatment of organic pollutants in water matrices include TiO_2_ in homogenously catalyzed process, while those use as heterogeneous catalysts; ZnO, SnO_2_, Al_2_O_3_, In_2_O_3_, ZnS, Fe_2_O_3_, CeO_2_, ZrO_2_, SiO_2_, CuO, MnO_2_ and CdS which function as sensitizers for light-induced redox processes. The TiO_2_ photocatalytic process leverage via direct photolysis of photon energy from UV and photocatalysis by producing hydroxyl radicals where TiO_2_ is photo-activated to generate hydroxyl radicals on the surface of the crystal before reaction with a wide range of organic pollutants which in turn lead to mineralization of the pollutants to CO_2_ and H_2_O UV radiation [125, 136]. The continuous design and development of various photocatalyst by researchers around the world affirmed that heterogeneous photocatalysis presents the best technological opportunities for the degradation of organic pollutants within the environment.

## Nanomaterial as photocatalyst in the degradation of PPCPs in WWTPs

Nanoparticle photocatalytic reactions can be described as the interaction of light energy with a metallic nanoparticle. This process is used in wastewater treatment as a result of their broad and high photocatalytic activities on the decomposition of various organic pollutants [137]. Nano-photocatalysts consist of nano-size order metal oxides, with semiconducting properties and strong ability to degrade large varieties of persistent organic pollutants such as dyes, detergents, pesticides, and volatile organic compound that WWTPs cannot remove [138, 139]. Quite several semiconductors such as ZnO, SrTiO_3_, Fe_2_O_3,_ SnO_2_, CuO_2_, WO_3,_ and Fe_3_O_4_ have been used for the decomposition of organic pollutants from different environmental matrices [139–141]. They can also be used for degrading halogenated and non-halogenated organic compounds, PPCPs, and heavy metals in some specific situations [7].

### Photocatalyst and photocatalytic method in advance oxidation procedures

The photocatalytic method is a promising technology used in the treatment of contaminants as a result of the ability of the catalyst to use sunlight as the energy source to initiate and facilitate the degradation of organic pollutants [142]. However, some of the challenges encountered with the use of photosensitive semiconductors as catalysts are low quantum efficiency, rapid recombination of photogenerated carriers, narrow optical response, and a slow transfer rate of electrons to oxygen which hinder their applications in the treatment of organic pollutants [143]. These limitations could be eliminated by employing strategies such as the modification of the textural properties, heterojunction construction, elemental doping, and morphology control to improve their photocatalytic performance [141].

The modification of photocatalysts that are highly active via visible light enhances the central part of the solar spectrum to be used. Therefore, to exploit the visible light during the photocatalytic process, there is a need for the usage of narrow band gap semiconductors like Cu_2_O, WO_3_, CdS, BiVO_4_ etc., for the degradation of different organic pollutants. Cheng et al. [144] reported the photocatalytic activity of Cu_2_O–Cu composites irradiated under visible light at 420 nm for the degradation of methylene blue, where 52% removal was achieved after 60 min. The intensity degraded after irradiation at 664 nm as observed by the disappearance of its peak after 60 min. Li et al. [145] synthesized Z-scheme heterostructure nanocomposites by loading mesoporous γ-Fe_2_O_3_ nanosphere on g-C_3_N_4_ nanosheet, the synthesized Z-scheme γ-Fe_2_O_3_/g-C_3_N_4_ heterostructure exhibits mesoporous features with the improved specific surface area providing reactive mass sites for tetracycline; also the constructed heterostructure which existed between γ-Fe_2_O_3_ and g-C_3_N_4_ efficiently extends the response range which leads to the quick transfer and separation of photoinduced charge carrier which enhances its photocatalytic activity for mineralization of tetracycline hydrochloride under visible light irradiation.

The working mechanisms behind the photo-catalyzed advanced oxidation process are based on the photoexcitation of electron in the catalyst. For instance, if photocatalyst, such as TiO_2,_ is irradiated with UV light, it causes a generation of holes (h^+^) and exits electron (e^−^) in the conduction band. In aqueous media, there is an entrapment of water molecules (H_2_O) onto the holes (h^+^), leading to the generation of hydroxyl radicals (·OH). These hydroxyl radicals are indiscriminate, highly reactive, non-selective, and powerful oxidizing agents, which in turn, oxidize organic pollutants into water and gaseous degradation products [137].

### TiO_2_ photodegradation process

TiO_2_ stands out as an excellent photocatalyst amongst the semiconductors that can be used for the degradation of PPCPs, due to its optical and electronic features, photosensitivity, physical and chemical stability, cost-effectiveness, resistance to light corrosion, non-toxicity and their high accessibility [146, 147]. Polymorphs of TiO_2_ include anatase, rutile, and brookite [148], of which anatase TiO_2_ have been shown to exhibit the highest photocatalytic activity as a result of the extended lifetime of the photoexcited electrons, and the fast migration of photogenerated electrons [149].

Literature reports indicated that the photocatalytic degradation of PPCPs using TiO_2_ as catalysts could result in mineralization reaching close to 99% within 1 to 3 h under various parameters. For example, the photocatalytic degradation of ofloxacin and atenolol irradiated under a UV lamp was almost complete after 240 min [60, 150]. Furthermore, photocatalytic mineralization of tetracyclines, paracetamol, caffeine, and atenolol as single/lone pollutants and in mixtures was achieved via TiO_2_ irradiated under UV and simulated solar irradiation, where the removal of tetracyclines reaches 90% after 35 min and that of paracetamol, caffeine, and atenolol were between 80 and 90% after 6 h, while the degradation of the mixture of these pharmaceuticals was achieved at 60% after 6 h under the same conditions [151]. He et al. [152] reported that 100%, 100%, 76%, and 74% photocatalytic degradation of propranolol, diclofenac, carbamazepine, and ibuprofen, respectively, was achieved after 96 h via the immobilization of TiO_2_ on the sand. The degradations were made possible as a result of the photo-oxidation of substrates from the single oxygen generated in the reaction mixture [153].

Considerable removal performance can be achieved in the presence of simulated solar light. Irradiated TiO_2_ was also applied for the mineralization of organic molecules such as acetylene in the gas phase. Acetylene mineralization/degradation in the gas phase follows a first-order kinetic in which no organic intermediate was detectable, suggesting that no pollutants or any intermediate species interfere in the photocatalytic mineralization [154]. However, the usage of TiO_2_ as a photocatalyst has critical limitations such as its wide intrinsic band gap that is 3.0 eV and 3.3 eV for rutile and anatase, respectively, and this results in achieving an activation at ultraviolet radiation at λ < 380 nm. This wide band gap can be reduced via doping of TiO_2_, in order to cause a shift in absorption in the visible light region of λ > 400 nm [116, 155]. Another challenge of TiO_2_ as a conventional powdered catalyst for photodegradation is the difficulty of their separation from the slurry of the reactor systems after photocatalysis, and this leads to low efficiency in photocatalytic activities over a range of application [125, 156].

Due to surface limitations and inadequate adsorption capacity of TiO_2_ resulting from non-porous nature, current studies are directed towards incorporating photocatalysts on porous supports in order to facilitate the efficient absorption of target environmental pollutants prior to subsequent oxidation aided by the photocatalyst. To achieve a more rapid and efficient removal of PPCPs in WWTPs, there is a need to modify the photocatalytic process through immobilization of TiO_2_ onto a suitable solid support such as activated carbon, molecular sieve, graphene, and minerals as a result of their ability to enrich the pollutants and improves their photocatalytic degradation rate due to its stability, mechanical resistance, high surface area and appropriate porosity [157, 158].

### ZnO photodegradation process

ZnO, with strong oxidation ability and a tremendous photocatalytic property, has also been used in the degradation of various organic pollutants due to its direct and broad band-gap energy close to UV spectral region, hence their thermal stability and considerable free-exciton binding energy. It is also relatively cheap when compared to TiO_2,_ which is said to be non-economical for the treatment of water on a large scale [159, 160]. ZnO nanoparticles have been reported to be more catalytically active compared to TiO_2_ for degradation of pharmaceuticals, where the photocatalytic degradation of 14 pharmaceuticals was studied using ZnO and TiO_2_ nanoparticles [161]. Study results showed that 95% removal was achieved after 40 min under UVA irradiation via ZnO nanoparticles catalyzed process, whereas only 40% removal was achieved for TiO_2_ catalyzed process under the same condition. The photocatalytic activities of different types of ZnO were studied for the degradation of methylene blue, where the reaction was monitored at pH 7.5 for 60 min under UV irradiation of 664 nm. ZnO nanofibers were noted to be the best among the fabricated ZnO, as photo-degradation reaches almost 100% for methylene blue removal [160]. The photocatalytic efficiency of ZnO/g–C_3_N_4_ composites was investigated by Ismael [162] for the degradation of methyl orange and 4-chlorophenol under visible light radiation. Its photocatalytic performance was due to the synergistic effects between ZnO and g-C_3_N_4,_ resulting in the interfacial transfer of photogenerated electrons and holes hence its effective charge separation resulting in their ability to be able to degrade methyl orange and 4-chlorophenol.

### WO_3_ photodegradation process

Another semiconductor which has been used in the photocatalytic degradation of organic pollutants is WO_3._ WO_3_ is an n-type semiconductor with high stability and a wide tunable band gap of around 2.6 eV, hence their application in the photocatalytic degradation of various organic pollutants. For instance, complete degradation of rhodamine B was achieved when WO_3_ nanoplatelet was employed, with the intensity peak of Rhb disappearing after 350 min, when the catalyst reacted with the organic pollutants to form an unstable intermediate under UV light irradiated at 554 nm [163]. Chen et al. [164] reported the fabrication of WO_3_/g-C_3_N_4_ nanocomposites by a simple direct precipitation method, and the Z-scheme photocatalyst WO_3_/g-C_3_N_4_ was designed and prepared by dispersing WO_3_ nanosheets on g-C_3_N_4_ nanosheets. The as-prepared WO_3_/g-C_3_N_4_ showed enhanced photocatalytic activity toward degrading methyl orange. The improved photocatalytic performance of WO_3_ nanosheet doped on g-C_3_N_4_ was due to the coupling of effect of g-C_3_N_4_ on WO_3_ resulting in a slight shifts to lower binding energy, which hints the strong interaction between WO_3_ and g-C_3_N_4_.

### Ag/AgCl/AC photodegradation process

Photocatalytic degradation involving tetracyclines reaches 97.3% in 60 min under visible light radiation through the use of Ag/AgCl/AC composites. The reactive species that aided the degradation are electron holes and superoxide radicals, while the exposed activated carbon surface in the composites photocatalyst enhances the tetracyclines adsorption onto its surface, thereby increasing the contact sites between tetracycline and photocatalyst which lead to a better and efficient photodegradation rate [165].

## Carbon-solid supports in photocatalytic degradation

The photocatalytic performance of various photocatalysts has been mostly enhanced for the degradation of organic pollutants by combining them with carbonaceous materials. These carbonaceous materials, when compared to single semiconductors, give additional advantages such as chemical stability, thermal stability, high surface area, pore structure, good conductivity, excellent electronic properties, better photocatalytic activity, activity under solar irradiation and easy separation [106]. Activated carbon (AC), carbon nanotubes CNTs) and graphene are among the best solid materials for use as catalyst support for photocatalytic purposes, especially those use for the degradation of PPCPs in water and wastewater, due to properties such as; large specific surface areas and high charge carrier mobility [166, 167]. Activated carbon is porous and amorphous solid carbon material that can be derived from coal or plants through carbonization at a temperature between 400 and 600 °C, with an activation process by use of steam or CO_2_ [168]. Activated carbon is non-photocatalytic in nature but can serve as a means to enhance the photocatalytic reaction between catalyst, e.g., TiO_2_ and the organic contaminants, by adsorption of pollutants on its surface to facilitate the degradation of organic contaminants [169].

The use of carbonaceous material in the degradation of PPCPs has been studied several times, and it has been proven that they can only adsorb pollutants, but cannot transform them, thus leading to the generation of hazardous products. In a situation where the adsorbents were not regenerated on-site, such adsorbents become hazardous waste and must be disposed of carefully, coming with additional cost. The use of carbonaceous material can, therefore, be expensive [7]. The affinity of solid materials for PPCPs results from the presence of functional groups such as carboxyl, carbonyl, phenol, lactone and quinone, which confers on them surface-active species and specific surface areas that attract solid materials and concentrating them close to the semiconducting active site for improved photodegradation [170]. This makes the composites serve as effective adsorbent material due to one or more combinations of either hydrophobic interaction, π–π interaction, hydrogen bonding interactions, and electrostatic and dispersion interaction [171]. The clogging of the pore on adsorbent material is also a common challenge.

### Activated carbon (AC) for photocatalytic degradation

Carbonaceous materials like activated carbon function as electron scavenger because of their enormous electron storage ability, which is known as Hoffman mechanism property [172]. They also act as a sensitizer-providing electron for semiconductor photocatalyst such as TiO_2_, g-C_3_N_4_, etc. The electrons are excited by photons of light energy, which produces the superoxide radicals from absorbed molecular oxygen. Moreover, the positively charged composite material attracts electron from semiconducting metal catalysts such as TiO_2_, to create a hole (*h*^+^) which interact with adsorbed water to generate hydroxyl radicals, in the TiO_2_ valence band. The presence of carbon–oxygen–titanium linkages within the composite materials leads to a reduction in the band gap and extends the absorption band into the lower energy visible range [116]. Moreover, the interest in the use of activated carbon as solid support for various photocatalyst semiconductor is as a result of its developed pore structures, very large surface area and high adsorption capacity, hence their usage as an adsorbent for both organic and inorganic pollutants [173].

Activated carbon in the TiO_2_ immobilized on activated carbon photocatalyst, serves as a reaction medium through which organic molecules are absorbed before degradation [174]. The complete removal of pharmaceuticals such as amoxicillin, ampicillin, acetaminophen, and diclofenac was achieved using AC–TiO_2_ composite material under sunlight irradiation for 180 min, and this cannot be achieved with bare TiO_2_ [173]. This is a result of the transfer of sorbate from activated carbon to photoactive TiO_2_ due to the presence of a common interface between them [167]. Another solid support that has been of great use owing to its high surface area, chemical and thermal stability is zeolite [175]. Their immobilization on TiO_2_ has proven to be efficient in the removal of highly volatile organic contaminants due to their adsorptive nature, and this can easily be supplied onto the TiO_2_ photocatalyst surface [176]. Bare TiO_2_ samples can rapidly deteriorate, while TiO_2_ composites can maintain a high adsorption efficiency to remove organic contaminants over a long period [177].

### Preparation of activated carbon and the fabrication of doped activated carbon

Khraisheh et al. [178] reported the preparation of activated carbon from coconut shell material, which is an inexpensive source of carbon. AC prepared from many other biomass materials including, plant remains, animal remains, agricultural wastes, decomposing materials, etc., have been reported [179–181]. Carbon sources for the preparation of activated carbon vary from chemical to biological/natural sources, while the method of preparation depends on the nature and characteristics of the carbon source. However, production using biological/natural material sources are relatively cheaper than chemical raw materials because the organic/natural sources are often waste materials, hence the need for the use of plant material as a source for activated carbon.

This technique is followed by the immobilization/doping of activated carbon with a metal oxide or nano metal oxides to form a composite catalyst. Different physical and chemical characteristics and attributes are achieved depending on how and which method is used for activated carbon preparation and how the metal oxide is doped/immobilized on the ACs. Arana et al. [182] reported that the reaction of TiO_2_ with activated carbon at different proportions showed that activated carbon not only increase the surface area but there is a modification of the acid–base properties and the UV spectrum of TiO_2_. The following methods have been used in the preparation of AC as a carbon source for photocatalytic degradation of organic pollutants: sol–gel, chemical vapor deposition, impregnation, pyrolysis, precipitation, hydrothermal preparation, microwave-assisted synthesis and sonochemical treatment [183–185].

The mostly used among the above method is the sol–gel method because there is an ultimate mixing or chemical interaction between activated carbon and the corresponding semiconductor photocatalyst. Studies proved that doped/immobilized metal oxides composite material possess superior quality and has the highest removal efficiency under UVC intensity. In TiO_2_ composite material, for example, the composite solid support doped with TiO_2_ showed higher photocatalytic activity, adsorption capacity, electron scavenging, and sensitization ability and extended visible light adsorption when compared to bare TiO_2_ [186]. The photocatalytic activity of prepared nanocomposite ZnO/AC where the activated carbon was prepared from biomass degrades more than 95% organic pollutants as a result of the addition of electrolyte, although this decreases the photoactivity of the catalyst [187].

### Carbon nanotubes doped composites in photodegradation

Carbon nanotubes are made up of cylindrical graphene sheets and can be classified either as single-walled carbon nanotubes (SWCNTs) and multi-walled carbon nanotubes (MWCNTs) [13]. CNTs possess a high specific surface area to mass ratio ranging between 75 and 1020 m^2^/g coupled with an exceptional sorption capacity [11, 188, 189]. They exhibited hydrophobic interactions, van der Waals forces, and π–π stacking, leading to the formation of four different types of adsorption sites such as inner cavities, interstitial channels, external grooves, and outermost surfaces [190], making them a suitable solid material for photocatalytic degradation of PPCPs. CNTs have interesting properties such as large electron storage capacity, superior metallic conductivity, and light adsorption at a broad range wavelength, thereby making them a suitable solid material in the enhancement of the photocatalytic activity of TiO_2_ [172]. As monitored on high-performance liquid chromatography, the photocatalytic degradation of oxytetracyclines reaches 88.7% within 60 min when MWCNT/BiVO_4_ composites were utilized. The photocatalytic enhancement was made possible as a result of the synergistic effect that occurs between MWCNTs and BiVO_4,_ which increased the separation rate of photogenerated electron–hole pairs [191].

The usage of CNTs–TiO_2_ for the photocatalytic degradation of caffeine was as a result of good contact between CNTs and TiO_2,_ thereby causing a better enhancement in their photocatalytic activity [6]. CNTs–TiO_2_–N has shown to exhibit a high photocatalytic activity under visible light irradiation for the degradation of ibuprofen [192]. This feature can be attributed to the fact that CNTs can function as electron scavengers resulting in a good binding between CNTs, TiO_2,_ and N used. Modification of CNTs on g-C_3_N_4_/BiVO_4_ was evaluated for the degradation of phenol in the presence of solar light irradiation. The synergistic interaction between the heterostructure was aided by H_2_O_2_ as the oxidizing agents for 80.6% removal of phenol after 2 h, where the BiVO_4_ helps to promote a better optical absorption ability over the well-contacted structure within the heterojunction while the presence of CNTs in the heterostructure enhances a higher photocatalytic performance as a result of its ability as an electron bridge mediator which is responsible in facilitating the photogenerated charge carrier separation [193, 194].

The methods that are employed during its preparation are as follows; hydrothermal, hydrolysis, restrained hydrolysis, simple evaporation and drying, sol–gel, surfactant wrapping sol–gel, chemical vapour deposition, physical vapour deposition, electrophoretic deposition, electrostatic attraction, solvothermal and impregnation [183, 192].

### Graphene-based nanocomposites in photocatalysis

Graphene is made up of a two-dimensional sheet of carbon atoms that are connected by an sp^2^ bond consisting of an aromatic π electron system [195]. Graphene possesses the following properties; high electron mobility, high mechanical strength, high thermal stability, and high specific surface area [196]. These unique properties made graphene have a higher density of potential adsorption sites, which are; open-up surface, a longitudinally-parallel external surface, and interstitial channels [197]. The relevance of graphene includes applications such as in sensors, energy conversion and storage, polymer composite, drug delivery systems, and environmental remediations [198–200]. There are different type of graphene that is available, which are; graphene oxide, reduced graphene oxide, fullerene, carbon nano-onion, and carbon nanotubes [201].

Graphene oxide (GO) and reduced graphene oxide (rGO) are the most commonly used graphene because they show a high efficiency during water treatment. These abilities to adsorb pollutants are due to the presence of several surface functional groups like hydroxyl, carboxyl, and epoxy that function as adsorption sites that aid the removal of contaminants from the water system [202, 203]. Graphene oxide (GO) is prepared via chemical functionalization of graphene through the following method; Brodie [204], Staudenmaier, Hoffman, and Hummer methods [205]. Graphene oxide (GO) is photocatalytically active and can function as an electric insulator as a result of the chemical disruption of the π network. Their electrical conductivity can be restored during the conversion of graphene oxide to reduced graphene oxide [206]. Reduced graphene oxide can be prepared using the chemical treatment [207], thermal mediation [208], and electrochemical treatment [209]. Both graphene oxide and reduced graphene oxide can be used for the photocatalytic degradation of PPCPs. GO–TiO_2_ can be prepared via the following method; solution mixing, sol–gel, hydrothermal, electrostatic self-assembly, molecular grafting, chemical exfoliation, liquid phase deposition, and electrospinning [95, 210, 211].

The choice of the method to use will be based on which of them shows a stronger interaction between graphene and the corresponding semiconductor photocatalyst, which in turn will enhance their photocatalytic activity. The degradation of Rhodamine reached 97.2% when rGO/g-C_3_N_4_ composite was employed. The efficiency of the composites was as a result of the synergistic effect of rGO/g-C_3_N_4_ heterostructure formed in the formation of coupled heterointerface and heterojunctions which efficiently and effectively aid in the transfer of photogenerated electron–holes pairs under the influence of visible light where photo-induced electrons can freely migrate along with the conductive network of rGO thereby preventing the recombination of the photogenerated carriers [212]. There is a presence of strong electronic and physical coupling when TiO_2_ nanosheet-graphene of 2D–2D composite interact, causing an enhancement of electron transfer between them, thus making the photocatalyst superior when used in the removal of 2,4-dichlorophenol as compared to TiO_2_. This is because of the availability of rich oxygen functional groups on the 2D surface of rGO, which enhances the ability of rGO to interact with other organic/inorganic compounds to form hybrid composites [213].

Shanavas et al. [213] reported that the advantage in the use of 2D/3D/2D heterojunction in the photocatalytic degradation of pharmaceuticals such as tetracyclines and ciprofloxacin is the reduction of photoexcited charge carrier migration distance for effective suppression of electron–holes pairs recombination, therefore, creating numerous high-speed photo-induced charge transfer nanochannels in rGO/Fe_2_O_3_/g-C_3_N_4_ nanocomposite by degrading tetracyclines and ciprofloxacin under visible light irradiation. Also, porous 3D rGO–TiO_2_ aerogel has been reported to remove over 99% of carbamazepine. This was made possible because the macro-porous 3D structure of the aerogel has abundant surface sites, effective charge separation causing an improvement in the mass transportation of the contaminants, thereby leading to easy separation of the catalyst after degrading the contaminants [214].

The excellent outcome experienced during the degradation of carbamazepine, ibuprofen, and sulfamethoxazole, which is up to 80% during removal and high durability in 45 h operation, is as a result of the immobilization of rGO on TiO_2_ on optical fibers [215]. However, since the photocatalytic activity of solid material immobilized on TiO_2_ has proved to be efficient in the degradation of PPCPs, there is, therefore, a need to develop a standard method with a pilot-scale experiment to ascertain the cost on a large scale use. Due to the photocatalytic activity of graphene–TiO_2_ composite, hence their usage in the degradation of PPCPs but their drawback in water treatment arises from the difficulty in separating and recovery of the catalyst after use.

## Catalyst preparation methods

The preparation of different photocatalysts can be achieved through various chemical processes. The synthesis methods are usually tailored to suit the size and morphology of the intended materials. Some simple synthetic techniques that have been used for the synthesis of functional photocatalyst materials are stated below.

### Coprecipitation

The coprecipitation method is a very facile and convenient method used in the synthesis of various photocatalysts. This method involves the dissolution of the salt precursor of interest into an aqueous solution followed by precipitation by addition of a base such as NaOH, KOH. This procedure is usually followed by washing and drying, then calcination of the precipitate at a desirable temperature to obtain the intended products [216]. The particle size, morphology, and composition of the synthesized photocatalyst prepared through this method can be controlled by adjusting different experimental parameters, such as the starting material, their ratio, the surface ligand, the reaction temperature with time, and the pH. Moreover, the size distribution of the as-prepared photocatalyst can be improved by the addition of ligands, such as surfactants, inorganic molecules, and polymers into the reaction medium, however, size control is generally poor for this method [217].

### Hydrothermal/solvothermal method

This method involves the chemical reaction where the precursors are dissolved in a solvent such as water, organic solvents, or KOH. The aqueous mixture will then be subjected to heat at a high temperature around 200 °C in a sealed stainless steel autoclave. Consequently, the pressure within the reaction autoclave is drastically increased above atmospheric pressure resulting in the generation of high crystalline materials without the need for further treatments [218]. The drawback of this method is its low yield of products when compared with the coprecipitation method.

### Thermal decomposition method

Thermal decomposition is an innovative method to synthesize stable nanoparticles. This method involves the thermolysis of organometallic complex precursors in high-boiling-point organic solvents in the presence of surfactants to synthesize nanoparticles of various materials. It is faster, clearer, and economical when compared with other methods [219]. It is also one of the easiest and the most convenient method used to synthesize monodispersed metal nanoparticles. Furthermore, it answers the greatest challenge of obtaining a controlled nanometric size and shape in nanotechnology research, which is achieved by controlling the concentrations of the precursors and surfactants; the solvent; and the experimental parameters such as heating rate, heating temperature, and heating time are usually regulated [217]. However, photocatalyst prepared by this method is usually coated with hydrophobic ligands, thereby making it insoluble in water. Therefore, a further surface modification step is required to render these nanoparticles water-soluble for efficient photocatalytic applications.

### Sol–gel method

The sol–gel method for the synthesis of nanomaterials is a convenient and versatile reaction technique in the synthesis of numerous functional photocatalyst. The sol–gel process is based on hydrolysis and polycondensation reaction involving the addition of a complexing agent such as; a polymer, citric acid, or other suitable organic entities into a solution of a colloidal dispersion of the metal ions of the target material. These complexing agents function as cross-linkers between the ionic substances in solution followed by gradual removal of the solvent molecules from the mixture to form a thick gel. The gel is then dried and pre-calcined to obtain the as-prepared powder. The as-prepared powders are then annealed at higher temperatures to obtain the finished product. Metal oxides can also be used as starting materials in this method [220, 221].

Several parameters, such as type of precursor, type of solvent, water content, pH, the concentration of precursor and temperature, can influence the structure of the initial gel, which in turn influenced the properties of the resulting materials, including the crystal structure, particle size, shape and crystallinity [222, 223]. The sol–gel method possesses many advantages. For instance, it allows tailoring of both the bulk properties, including phase composition and surface characteristics such as the surface area, the total pore volume distribution, etc., of a material on a nanometer scale from the earliest stages of processing. The main drawbacks are in the possible high cost for the majority of alkoxide precursors, and processing steps are long.

## The use of graphitic carbon nitride (g-C_3_N_4_) as an efficient catalyst for the degradation of pharmaceuticals

**Fig. 3:**
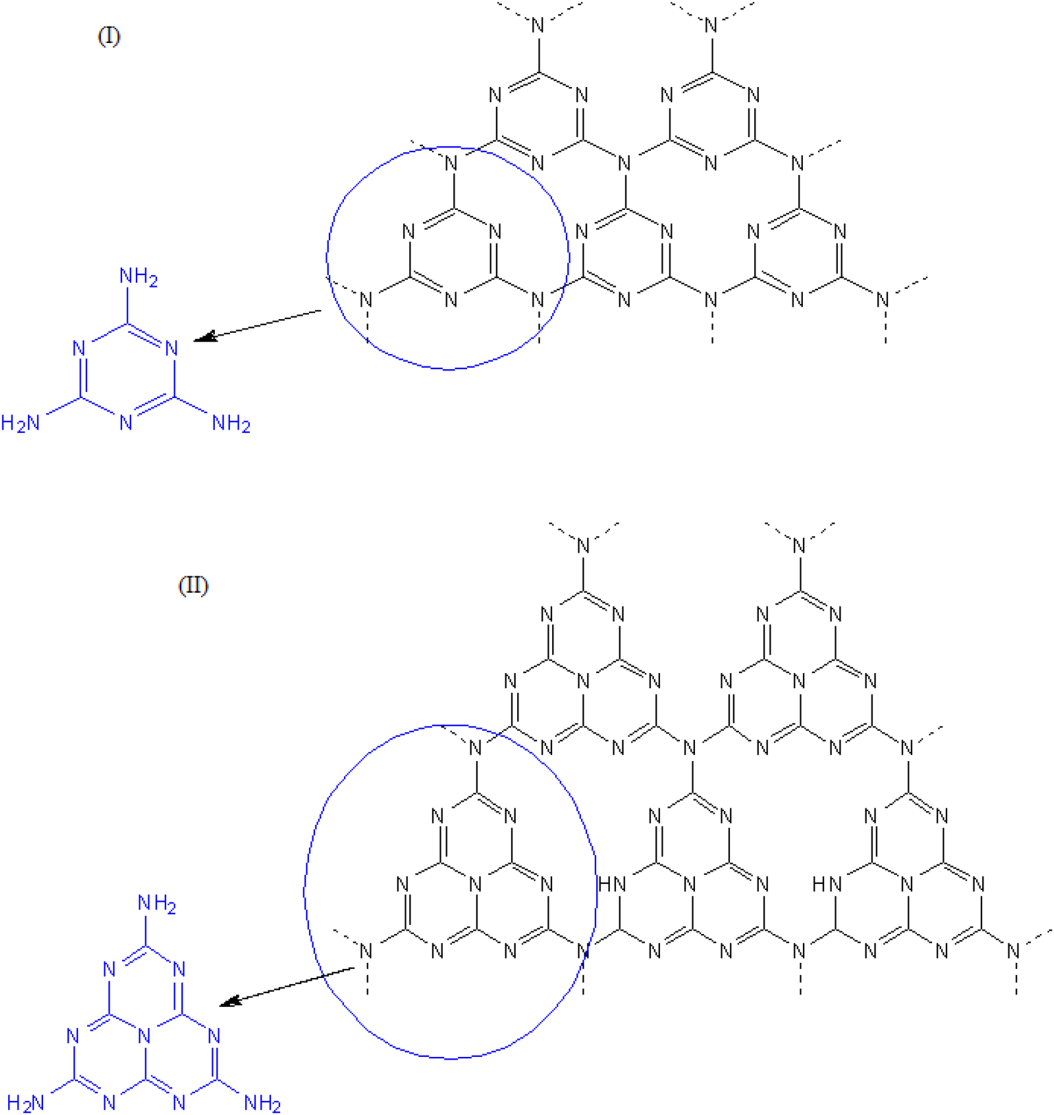
**I** s-triazine and **II** tris-s-triazine based structures of g-C_3_N_4_ [227]

The large band gap energy of semiconductor photocatalysts such as TiO_2_ (3.2 eV), ZnO_2_ (3.37 eV), constitute a significant limitation to their photocatalytic usage under visible light spectrum due to its low utilization of solar energy. This limitation makes this method an expensive means for the photocatalytic process; hence the urgent need to research more on visible light semiconductor photocatalyst. g-C_3_N_4_ has emerged one of the next generation photocatalysts due to its cheap synthesis, fascinating electronic band structure, non-toxic, graphene-like two-dimensional structure coupled with good visible light adsorption ability and chemical stability for removal of hazardous pollutants from the environment and clean energy production [224, 225]. They are made up of two main units to establish their allotropic nature which are; tri-s-trianzine/heptazine (C_6_N_7_) and s-triazine (C_3_N_3_) rings (Fig. 3); where the tri-s-triazine based g-C_3_N_4_ is highly favoured and the most stable phase of g-C_3_N_4_ thermodynamically [226].

Since the discovery of g-C_3_N_4_ by Wang et al. [225] as a promising visible light photocatalyst for the evolution of H_2_, much effort has been directed towards its synthesis. g-C_3_N_4_ can be prepared by thermal polymerization using the following precursors based on different reaction conditions and differences in the material degrees of condensation; cyanamide, dicyandiamide, melamine, urea, thiourea and ammonium thiocyanate [227–229]. g-C_3_N_4_ has a low energy band gap between 2.7 and 2.8 eV as a result of the presence of sp^2^-hybridized carbon and nitrogen forming the π-conjugated electronic structure, possess excellent chemical stability, does not dissolve in acid, alkali or organic solvents making it to be environmentally friendly material [229, 230].

However, their application in environmental remediations are limited by several obstacles such as low specific surface area, the high recombination rate of charge carrier and low electrical conductivity, hence the need to develop a modified g-C_3_N_4_-based heterojunction photocatalyst with improved physicochemical properties that can be applied in the degradation of organic pollutants in the environment [231, 232]. The modification can be achieved through exfoliation of bulk g-C_3_N_4_ into nanosheet, nanotubes and quantum dots, deposition of noble metal, doping with metal, coupling with other semiconductors such as TiO_2_, ZnO, Ag_2_O, MoS_2_, incorporating with carbonaceous materials like; activated carbon, graphene oxides, reduced graphene oxide, carbon nanotubes, and many others to form nanocomposites [233–235].

### Modification of g-C_3_N_4_

To overcome the drawback in g-C_3_N_4_ associated with their photocatalytic application, specific surface area, charge separation, and opticals for g-C_3_N_4_ have been improved. Numerous heterostructure based g-C_3_N_4_ photocatalyst has been prepared by many researchers with improved suitable band structures, shaped at the interfaces between g-C_3_N_4_ and metal particles, inorganic semiconductors or carbonaceous materials have shown to enhance their charge carrier separation leading to its improvement in their photodegradation activity [236–238]. Hence, the interest in the synthesis of heterostructure based g-C_3_N_4_ with suitable band position resulting in the suppression of the recombination rate of the photogenerated electron–hole pairs for improved photocatalytic degradation of hazardous contaminants from the environments.

Fabrication of g-C_3_N_4_ based semiconductor photocatalyst is considered as one of the effective and more feasible strategies to enhance the photocatalytic activity of g-C_3_N_4_ where the formation of a heterojunction system improve the photo-generated electron–hole pairs separation leading to the improvement in their photocatalytic activity as seen in the following g-C_3_N_4_ based photocatalyst g-C_3_N_4_/TiO_2_, g-C_3_N_4_/WO_3_, g-C_3_N_4_/CdS, g-C_3_N_4_/Cu_2_O, g-C_3_N_4_/CdS/Cu_2_O for the degradation of numerous organic pollutants in the environment [239–241].

Chen et al. [242] synthesized a series of visible-light-driven MgIn_2_S_4_/g-C_3_N_4_ heterostructures for the degradation of 4-nitroaniline and methyl orange. The improvement in the photocatalytic activity of MgIn_2_S_4_/g-C_3_N_4_ can be attributed to the faster charge separation and transport that existed between the interfaces and band structures of MgIn_2_S_4_ nanoplates and g-C_3_N_4_ nanosheets making it an efficient visible-light-driven composite for the degradation of hazardous pollutants. The photocatalytic performance of Z-scheme g-C_3_N_4_/CdS composites for the degradation of erythromycin and tetracycline was remarkably enhanced as compared to that of pure g-C_3_N_4_ and CdS owing to the perfect matching of the band gap, the interface of the close photocatalyst was lower than that of CdS which give rise to the formation of heterojunction with g-C_3_N_4_ which suppresses the photo corrosion of CdS [243].

Table 2 below shows how different heterostructure composites have been used for the degradation of various organic pollutants.

**Table 2:** Different synthesized heterostructure composites for the degradation of various organic pollutants

Photocatalyst	Synthesis method	Pollutants	Removal efficiency (%)	Time	Light source	References
g-C_3_N_4_	Polycondensation	Ibuprofen	20	4 h	35 W Xenon lamp	[244]
		Aspirin	30			
		Ciprofloxaci n	60			
		Tetracycline	86			
BaTiO_3_/TiO_2_	One-step calcination	Acetaminophen	95	4 h	500 W Xenon lamp	[245]
ZnO/g-C_3_N_4_	Thermal condensation	Tetracycline	78.4	50 min	300 W Xenon lamp	[246]
		Oxytetracycline	63.5			
Ag/g-C_3_N_4_	Photo-reduction	Sulfamethazole	99.5	60 min	300 W Xenon lamp	[247]
g-C_3_N_4_/TiO_2_/Fe_3_O_4_@SiO_2_	Sol–gel	Ibuprofen	92	3 h	330 Wm^−2^ compart fluorescent lamps	[248]
g-C_3_N_4_/MoS_2_	Hydrothermal	Levofloxaci n	75.81	140 min	Ultrasonic wave at 30 kHz	[234]
		Methylene Blue	98.43	14 min		
Ag_3_PO_4_/GO/g-C_3_N_4_	Chemical precipitation	Rhodamine B	94.8	50 min	150 W Xenon lamp	[249]
BiVO4/TiO2/RGO	Hydrothermal	Tetracycline	96.2	60 min	1000 W Xenon lamp	[250]
		Oxytetracycline	97.5			
		Chlortetracycline	98.7			
		Doxycycline	99.6			
Ag_2_O/CeO_2_	Thermal deposition	Enrofloxacin	87.11	120	300 W Xenon lamp	[251]
g-C_3_N_4_/CNT/BiVO_4_	Wet impregnation	Phenol	80.6	120 min	Sunlight (illumination intensity; 500 W halogen lamp)	[192]
g-C_3_N_4_/AgBr/rGO	Hydrothermal reaction	Tetracycline	78.4	90 min	250 W Xenon lamp	[252]
		2,4-Dichlorophenol	68.2	6 h		
rGO/WS_2_/Mg-doped ZnO	Electrostatic self-assembly and coprecipitation	Rhodamine B	95	14 min	UV lamp	[253]
			90	90 min	Solar light	

### Mechanism of g-C_3_N_4_ photocatalyst

The absorption of photon energy by g-C_3_N_4_ photocatalyst, which is either equal or greater than the band energy of g-C_3_N_4_ leads to the generation of electrons (e^−^) in the valence band (VB) to be excited before migrating to the conduction band (CB). The generated electron will be excited and leave the photo-generated holes (h^+^) in the valence band. Both the photoexcited electron (e^−^) and photogenerated holes (h^+^) will be trapped at the surface of the catalyst where the h^+^ react with absorbed H_2_O to produce ·OH radicals while e^−^ react with absorbed O_2_ to produce $\cdot{\text{O}}_{{2}}^{ - }$ radicals before reacting with organic pollutants and finally degrade them [254, 255]. The mechanism is summarized below;

$$ {\text{g-C}}_{3} {\text{N}}_{4} \mathop{\longrightarrow}\limits^{{{\text{h}}\upnu }}{\text{e}}^{ - } \;{}_{{{\text{CB}}}}\left( {{\text{g-C}}_{3} {\text{N}}_{4} } \right) + {\text{h}}^{ + } \;{}_{{{\text{VB}}}}\left( {{\text{g-C}}_{3} {\text{N}}_{4} } \right) $$

$$ {\text{e}}^{ - }_{{{\text{CB}}}} \left( {{\text{g-C}}_{{3}} {\text{N}}_{{4}} } \right) \, + {\text{ O}}_{{2}} \to {}^{ \cdot }{\text{O}}_{{2}}^{ - } $$

$$ {\text{h}}^{ + }_{{{\text{VB}}}} \left( {{\text{g-C}}_{{3}} {\text{H}}_{{4}} } \right) \, + {\text{ OH}}^{ - } \to {}^{ \cdot }{\text{OH}} $$

$$ {}^{ \cdot }{\text{O}}_{2}^{ - } + {\text{Organic}}\;{\text{pollutants}} \to {\text{CO}}_{2} + {\text{H}}_{2} {\text{O}} $$

$$ {}^{ \cdot }{\text{OH}} + {\text{Organic}}\;{\text{pollutants}} \to {\text{CO}}_{2} + {\text{H}}_{2} {\text{O}}{.} $$

**Fig. 4:**
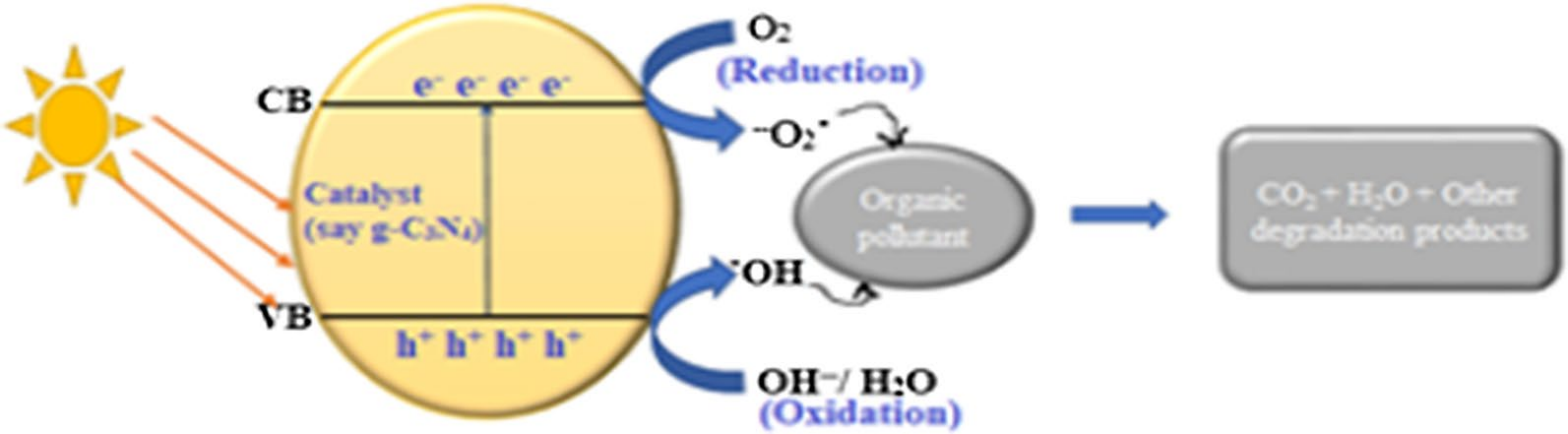
Schematic representation of photocatalysis mechanism (adapted with modification from [256])

However, in the photocatalytic process for the degradation of organic pollutants, it is crucial to understand the transformation of the different intermediates involved in photocatalysis as it will provide information about the toxicity of the intermediates as well as risk assessment. A schematic representation of photocatalysis mechanism is shown in Fig. 4.

Although many works have been done in fabricating a g-C_3_N_4_ based photocatalyst for environmental remediation, however, much effort needs to be directed towards the construction of g-C_3_N_4_-based heterostructure photocatalyst. This act would improve the separation of photogenerated electrons and inhibit the electron–hole pair recombination with an excellent possibility for an efficient and effective photocatalytic activity for the degradation of pharmaceuticals under visible light irradiation.

## Conclusion

In conclusion, this review highlighted a detailed overview of the detection of organic pollutants such as pharmaceuticals and personal care products, endocrine-disrupting chemicals, organic dyes, and various industrials waste in environmental matrices. Additionally, it was revealed that most researches focus on the occurrence of the parent pharmaceutical compound while the transformation products which may even be more toxic than the parent drugs are ignored, hence more focus is needed on how the chronic exposure to these contaminants affect humans and other ecosystems, particularly the non-target organisms. The environmental impacts of these emerging contaminants led to the need for the development of a technology for its removal from the environment. This problem of pharmaceutical contamination of the environment could be attributed to the inability of the wastewater treatment plants to completely remove the contaminants, making them one of the common sources through which these pharmaceuticals get into the environment. Even though various methods such as activated sludge, wetlands, adsorption, membrane processes have been developed for the removal of these pollutants, unfortunately, many research reports have proven that these methods are ineffective in removing these contaminants. Hence, the advanced oxidation process using a photocatalytic method becomes relevant. In this method, various semiconducting photocatalyst can be immobilized on carbonaceous materials, metal oxides, or another semiconductor as the best alternative for the degradation and mineralization of these organic pollutants into harmless products and at the same time, the catalyst can still be recovered and recycled.

Moreover, it should be noted that from the various reports reviewed, photocatalyst materials emerged as an efficient and effective technology for the degradation of various organic pollutants ranging from pharmaceutical and personal care products to different dyes. Furthermore, the engineering of metal-free carbon-based photocatalyst holds a great advantage as an efficient and effective means in degrading organic pollutants because of their tunable band gap, visible light responses, the formation of complex structures, excellent chemical stability, economical, reusability, and ease of fabrication. However, despite the significant progress made in the synthesis of photocatalyst for environmental remediation, their photocatalytic performance for degradation and mineralization of pharmaceutical and personal care products with their intermediates still need additional improvement for practical application. Also, their activities are limited by insufficient light absorption, rapid electron–hole pair’s recombination rate, difficulty in the separation and recycle of photocatalysts, and photo-corrosion of some semiconductor photocatalysts. The process might be complicated at times, costly, and time-consuming, hence the need to develop a method that is more efficient, easy, affordable, and environmentally friendly for the removal of organic pollutants at the WWTPs. Thus, the need for the green synthetic routes for more novel based heterostructure photocatalyst. In addition, the synergistic effects that existed within the heterostructure photocatalyst, which aid their photocatalytic activity, need more investigation. The mechanism of the degradation pathways, also, needs much attention to be able to verify the most active reactive species in photodegradation of organic pollutants. Finally, this review tends to serve as a great advantage for material scientists and nanotechnologists who are interested in seeking materials for environmental degradation of organic pollutants.

## Data Availability

All the information and data cited in this review were generated from literature research and listed in the references. The information, datasets used and analyzed during the current study are available from the corresponding author on reasonable request. We have presented all data in the form of tables and figures.

## Abbreviations

AC: Activated carbon
AOPs: Advanced oxidation processes
CB: Conduction band
CNTs: Carbon nanotubes
COD: Chemical oxygen demand
DCF: Diclofenac
EPA: Environmental Prevention Agency
eV: Band gap energy
GCMS: Gas chromatography mass spectrometry
GO: Graphene oxide
LCMS: Liquid chromatography mass spectrometry
LOD: Below limit of detection
LOQ: Below limit of quantification
NSAIDs: Non-steroidal anti-inflammatory drugs
PPCPs: Pharmaceuticals and personal care products
RGO: Reduced graphene oxide
UHPLC: Ultra-high-performance liquid chromatography
UV: Ultraviolet light
VB: Valence bond
WWTPs: Wastewater treatment plant
